# Investigating form and content of emotional and non-emotional laughing

**DOI:** 10.1093/cercor/bhac334

**Published:** 2022-09-09

**Authors:** Giada Lombardi, Marzio Gerbella, Massimo Marchi, Alessandra Sciutti, Giacomo Rizzolatti, Giuseppe Di Cesare

**Affiliations:** Italian Institute of Technology, Cognitive Architecture for Collaborative Technologies Unit, via Melen 83, 16152 Genova, Italy; Department of Informatics, Bioengineering, Robotics and Systems Engineering (DIBRIS), University of Genoa, via dell'Opera Pia, 16145 Genova, Italy; Department of Medicine and Surgery, University of Parma, via Volturno 39/E, 43125 Parma, Italy; Department of Computer Science, University of Milan, via Comelico 39, 20135 Milano, Italy; Italian Institute of Technology, Cognitive Architecture for Collaborative Technologies Unit, via Melen 83, 16152 Genova, Italy; Istituto di Neuroscienze, Consiglio Nazionale delle Ricerche, via Volturno 39/E, 43125 Parma, Italy; Italian Institute of Technology, Cognitive Architecture for Collaborative Technologies Unit, via Melen 83, 16152 Genova, Italy

**Keywords:** laughing, fMRI, insula, vitality forms, emotion

## Abstract

As cold actions (i.e. actions devoid of an emotional content), also emotions are expressed with different vitality forms. For example, when an individual experiences a positive emotion, such as laughing as expression of happiness, this emotion can be conveyed to others by different intensities of face expressions and body postures. In the present study, we investigated whether the observation of emotions, expressed with different vitality forms, activates the same neural structures as those involved in cold action vitality forms processing. To this purpose, we carried out a functional magnetic resonance imaging study in which participants were tested in 2 conditions: emotional and non-emotional laughing both conveying different vitality forms. There are 3 main results. First, the observation of emotional and non-emotional laughing conveying different vitality forms activates the insula. Second, the observation of emotional laughing activates a series of subcortical structures known to be related to emotions. Furthermore, a region of interest analysis carried out in these structures reveals a significant modulation of the blood-oxygen-leveldependent (BOLD) signal during the processing of different vitality forms exclusively in the right amygdala, right anterior thalamus/hypothalamus, and periaqueductal gray. Third, in a subsequent electromyography study, we found a correlation between the zygomatic muscles activity and BOLD signal in the right amygdala only.

## Introduction

It is well established that, in both transitive and intransitive motor behaviors, there are 2 components: the content (i.e. the goal) and the form (i.e. how the goal is achieved). For example, grasping an object or passing it to another person may be executed gently or rudely, energetically or hesitantly. These actions forms have been named vitality forms by Stern (1985).

**Fig. 1:**
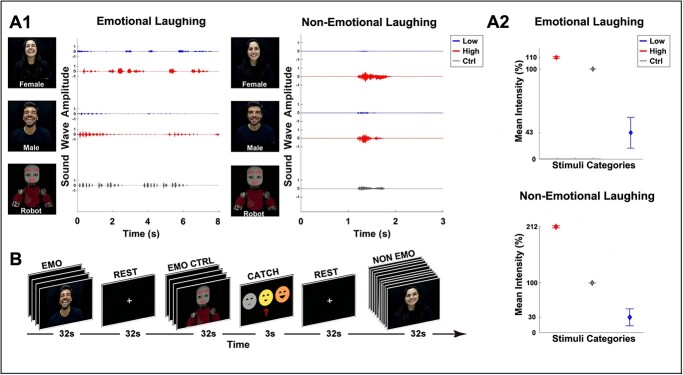
Physical properties of stimuli (A1–A2). Graph A1 shows the sound wave amplitude relative to an example of emotional and non-emotional laughing stimuli expressed by a male actor and a female actress with 2 different intensities (low, blue color; high red color) corresponding to 2 different vitality forms or by a robot (control, gray color). Graph A2 shows the mean intensity of the same stimuli. Experimental paradigm (B). For each condition, the experimental stimuli were presented in blocks of consecutive stimuli for a total duration of 32 s followed by a rest period lasting 32 s. In 20% of cases, participants were presented with catch trials (3 s) in which they were required to indicate the last stimulus observed (high, low, robotic).

Rizzolatti and Sinigaglia (2016) noticed that not only cold actions but also emotions are expressed with different vitality forms. For example, when an individual experiences negative emotions, such as disgust or anger, those can be expressed by a fleeting grimace or a contorted face. Similarly, a positive emotion, such as laughing, can be expressed by different face and body postures. It is important to note that, differently to other emotions, laughing can occur not only in emotional contexts but also in non-emotional ones. In particular, emotional laughing is typically associated to an activation of the orbicularis muscles in addition to the zygomatic ones (Duchenne smile) and to a subjective expression of mirth, commonly produced with the specific aim of promoting affiliation and social bonding (Dunbar et al. 2012). On the other hand, laughing plays also a strategic role in non-emotional contexts including verbal and non-verbal communication (Scott et al. 2014; Provine 2016). Such non-emotional laughing could be produced voluntary, like in a conversation, thus representing the “cold” counterpart of emotional laughing. Unlike the emotional laughing, the voluntary one shows an activation exclusively of the zygomatic muscles (non Duchenne smile). Note that, both emotional and non-emotional laughing can be expressed by a weak or overt laughing and a faint or strong smile, respectively, thus with different vitality forms. By using stimuli representing these 2 types of laughing (emotional and non-emotional), in the present study we aimed to assess if the observation of vitality forms expressed in emotional behavior involved the same neural substrates associated to cold actions (non-emotional). For this purpose, we carried out a functional magnetic resonance imaging (fMRI) experiment in which participants were tested in 2 main conditions. In the first condition (emotional laughing), participants observed and listened to an actor or an actress who spontaneously laughed with 2 intensities (i.e. high and low) corresponding to different vitality forms or, in the control condition, they observed and listened to a humanoid robot (iCub) laughing in a “robotic” neutral way (Fig. 1). In the second condition (non-emotional laughing), participants observed and listened to the same actors pronouncing the Italian verb “ridi” (English verb: “laugh”), with 2 different intensities (i.e. high and low) corresponding to different vitality forms and simultaneously smiling. In the control condition, they observed and listened to the humanoid robot pronouncing the same verb in a “robotic” neutral way (Fig. 1). Thus, the aim of the control condition was to allow participants to perceive the content of each stimulus (respectively the laughter and the command verb: “laugh”) without any vitality form information. During the experiment, participants were required to pay attention to stimuli discriminating different levels of vitality forms and were asked not to laugh to avoid activations related to the motor aspects of laughter.

In line with previous studies (Wild et al. 2003; Pohl et al. 2013), results of the fMRI experiment showed that the observation of both types of laughing produced the activation of regions known to be involved in the processing of facial expressions such as the superior temporal sulcus (STS), the ventral premotor cortex, the pre-supplementary motor area (pre-SMA) and the anterior-ventral sector of the insula. Furthermore, we observed that the observation of emotional laughing produced in addition, mainly on the right hemisphere, the activation of cortical and subcortical areas, such as the temporal pole, the amygdala, the periaqueductal gray, the habenula/medial pulvinar, and the anterior thalamus/hypothalamus. These findings are in line with the role of these structures in the processing of autonomic and visceral phenomena associated to the observation of facial expressions with an emotional content. Particularly, during the phenomenon of “emotional contagion” (Hatfield et al. 1993), the perception of a laughing face evokes in the viewer, immediately and unconsciously, corresponding facial reactions (facial muscles activity). In this regard, an interesting point we addressed was to assess whether the phenomenon of emotional contagion can be related to some of the neural structures found activated during the observation of emotional laughing.

For this purpose, we performed an electromyography (EMG) study to indirectly measure the association between brain responses and emotional motor activity on the same group of participants during the observation of the emotional laughing stimuli used in the fMRI experiment. Results of the EMG experiment showed a significant difference of the zygomatic muscles activation during the discrimination of the 2 vitality forms conveyed by the emotional laughing. Furthermore, we found a significant correlation between the EMG signal recorded from the zygomatic muscles and the BOLD activity of the right amygdala. This finding suggests that the amygdala is involved not only in the discrimination of emotional laughing but also in its expression during the emotional contagion phenomenon.

## Materials and methods

### Participants

Sixteen healthy right-handed volunteers (11 females and 5 males, mean age = 25.5, SD = 3.5) took part in the fMRI experiment. Due to the COVID-19 pandemic, 2 participants did not take part in the subsequent EMG study. The EMG study was carried out 2 months later the fMRI study. All participants had normal or corrected-to-normal vision and normal hearing. None of them reported cognitive or neurological disorders or current use of any psychoactive medications. All participants gave their written informed consent to participate in the experiment. The experiment was approved by the ethics committee of the University of Parma (552/2020/SPER/UNIPR) in accordance with the Declaration of Helsinki.

### fMRI study

#### Stimuli

In order to record emotional laughing stimuli, a male actor and female actress were required to observe a series of amusement video-clips. A video-camera recorded the natural reactions (laughing) of actors during the observation of these video-clips. Notably, video-clips showed people while spontaneously laughing with different intensities in several social contexts.

In contrast, in order to record the non-emotional laughing stimuli, the same actors pronounced the request Italian verb “ridi” (English verb: “laugh”), accompanied by a spontaneous smiling expression, as when an individual naturally interacts with somebody else. These requests were expressed with 2 intensities corresponding to different vitality forms (high and low).

During the video-recording, all vocal sounds produced by the 2 actors were recorded using a cardioid condenser microphone (RODE NT1) placed 20 cm from them and digitized with an A/D converter module with phantom power supply (M-AUDIO M-TRACK).

As a control, we video-recorded the humanoid robot iCub reproducing a neutral and robotic laughing and pronouncing the same request verb (“ridi”) expressing it in a flat way and without any vitality form (Fig. 1). This control voice was obtained by a vocal synthesizer (TextAloud software) and then processed with Cool Edit Pro software.

The physical characteristics of the vocal sounds were computed by using MATLAB software (The Mathworks, Natick, MA). Then, on the basis of sound amplitude and intensity (pitch) of each recorded video, we selected the best emotional and non-emotional laughing stimuli conveying high and low intensities corresponding to two vitality forms respectively (Fig. 1). Finally, a preliminary behavioral study was carried out to validate these stimuli (for details see Supplementary Material).

#### Experimental design

The experiment was presented as a blocked design (Fig. 1B). It was based on 4 different conditions: emotional laughing, non-emotional laughing, emotional laughing control, non-emotional laughing control. A total of 18 experimental stimuli were presented. In particular, 8 stimuli were shown for the emotional laughing condition (2 actors x 2 vitality forms x 2 laughs), 8 for the non-emotional laughing condition (2 actors x 2 vitality forms x 2 verbal requests) and 2 for the control conditions (1 emotional laughing control + 1 non-emotional laughing control). The experiment consisted in 3 functional runs with a total of 6 blocks for each condition. Each functional run lasted about 10 min. Finally, in order to identify the sector of the insula involved in execution of a simple smile, we also carried out a localizer run (for details see Supplementary Materials, Fig. S4).

#### Paradigm and task

Participants laid in the scanner in a dimly lit environment. The stimuli were presented via digital audio–video system (VisuaSTIM) with 30 dB noise-attenuating headset with 40 Hz to 40 kHz frequency response and with a 500,000 px x square inch resolution with horizontal eye field of 30°. The digital transmission of the signal to the scanner was via optic fiber. The software E-Prime 2 Professional was used both for stimuli presentation and the recording of participants’ answers. During stimuli presentation, participants were requested to fixate a white cross on the center of the black screen paying attention to the stimuli and were asked not to laugh to avoid activations related to the motor aspects of laughter. A block design was used in the experiment. Stimuli were presented in blocks of 4 (emo block) or 10 (non-emo block) consecutive stimuli presented in a random order. The total duration of each emo/non-emo block was 32 s. Each block was then followed by a rest period of 32 s (Fig. 1B). In the 20% of cases (catch trials), participants were asked to indicate, by pressing a button on a response box placed inside the scanner, the form of the last observed stimulus (high, low, robotic).

#### Statistical analysis

**Fig. 2:**
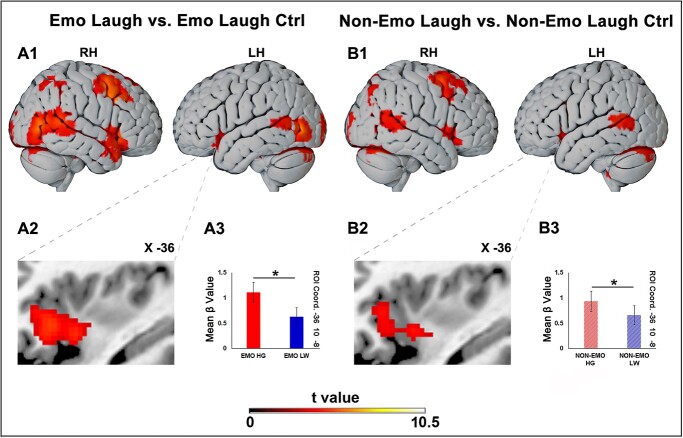
Brain activations resulting from the emotional laughing vs. emotional laughing control contrast (A1) and the non-emotional laughing vs. non-emotional laughing control contrast (B1). The selective activation of the left insula obtained in these 2 contrasts is shown in panels A2 and B2, respectively. ROI analysis carried out in the left insula (A3, B3). The horizontal lines above the columns indicate the comparisons between high and low vitality forms. LH, left hemisphere; RH, right hemisphere. These activations are rendered using a standard Montreal Neurological Institute Brain Template (PFWE < 0.05 at cluster level). *Significant differences (*P* ≤ 0.05).

**Fig. 3:**
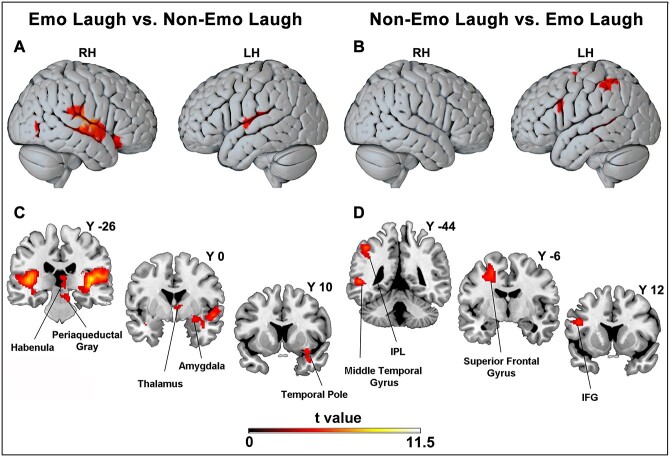
Cortical and subcortical activations enhanced by the emotional laughing vs. non-emotional laughing contrast (A-C) and the non-emotional laughing vs. emotional laughing contrast (B-D). These activations are rendered using a standard Montreal Neurological Institute Brain Template (PFWE < 0.05 at cluster level).

Data were analyzed using a random-effects model (Friston et al. 2002), implemented in a 2-level procedure. In the first level, single-subject fMRI BOLD signal was modeled by using a general linear model (GLM) comprising the following regressors: emotional laughing (Emo Laugh), non-emotional laughing (Non-Emo Laugh), emotional laughing control (Emo Laugh Ctrl), non-emotional laughing control (Non-Emo Laugh Ctrl), and response (Resp). Within each block, the videos were modeled as a whole event (duration 32 s). The catch trials, intermixed with experimental blocks, were modeled as events lasting 9 s (response 3 s; post-response 6 s). In the second level analysis (group-analysis), the BOLD signal was modeled by using a GLM comprising 4 regressors (Emo Laugh, Non-Emo Laugh, Emo Laugh Ctrl, Non-Emo Laugh Ctrl). This model enabled us to assess activations associated with each condition vs. implicit baseline (Supplementary Fig. S2) and activations resulting from the direct contrast between conditions (Emo Laugh vs. Emo Laugh Ctrl, Non-Emo Laugh vs. Non-Emo Laugh Ctrl, Fig. 2; Emo Laugh vs. Non-Emo Laugh, Non-Emo Laugh vs. Emo Laugh, Fig. 3). Furthermore, in brain regions highlighted by these contrasts, we also evaluated the BOLD activity during the processing of high and low vitality forms. For this purpose, single-subject fMRI BOLD signal was modeled by using the following regressors: emotional laughing high (Emo Laugh High), emotional laughing low (Emo Laugh Low), non-emotional laughing high (Non-Emo Laugh High), non-emotional laughing low (Non-Emo Laugh Low), emotional laughing control (Emo Laugh Ctrl), non-emotional laughing control (Non-Emo Laugh Ctrl), and response (Resp). In the second level analysis (group-analysis), the BOLD signal was modeled by using a GLM comprising 6 regressors (Emo Laugh High, Emo Laugh Low, Emo Laugh Ctrl, Non-Emo-Laugh High, Non-Emo Laugh Low, Non-Emo Laugh Ctrl).

### EMG study

#### Stimuli and experimental design

**Fig. 4:**
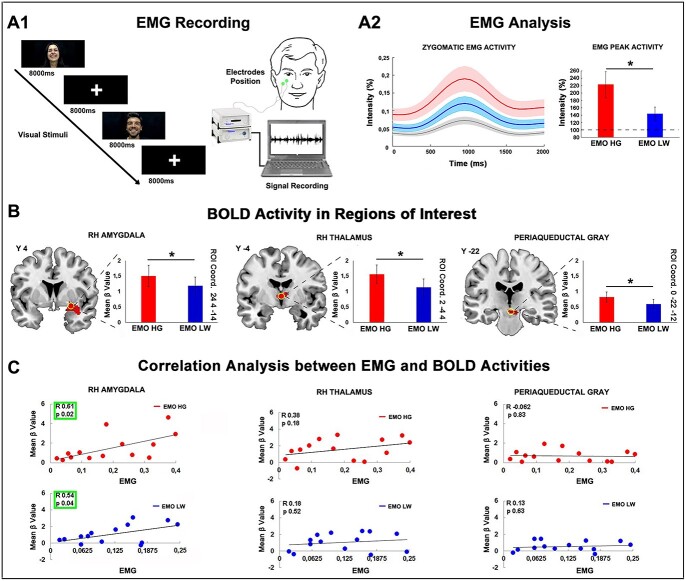
Experimental paradigm and setup used in the EMG study (A1). The graph shows the mean peak activity of zygomatic muscle recorded during the discrimination of emotional laughing stimuli conveying (high, red color; low, blue color) and not conveying (control, gray color) vitality forms (A2). The dashed line (100%) indicates the contagion effect obtained during the control condition. Error shadings indicate standard error of the mean. Contrast estimate (mean β value) in the right amygdala, in the right anterior thalamus and in the periaqueductal gray. Asterisks indicate significant difference between high- and low-vitality forms (*P* ≤ 0.05) (B). Correlation analysis between BOLD and EMG activities carried out in all 3 ROIs for both high and low vitality forms. The green boxes highlighted significant correlations (*P* < 0.05) (C).

During the EMG experiment, participants were presented with the emotional laughing stimuli presented during the fMRI study (Fig. 4A1). In particular, a total of 30 stimuli (each lasting 8 s) were shown in a randomize order (10 for the emotional laughing high, 10 for the emotional laughing low, and 10 for the emotional laughing control). Between stimuli, a rest period of 8 s was inserted to allow the EMG signal to return to the baseline.

#### Paradigm and task

Participants sat comfortably on a chair in front of a monitor. They were presented with videoclips showing the emotional laughing stimuli and were required, as occurred during the previous fMRI experiment, to pay attention to them. In the 20% of cases (catch trials), participants were asked to indicate with a mouse held in their right hand the form of the last stimulus perceived (high, low, robotic). Two electrodes were placed on the right zygomatic muscle of participants and a reference electrode (ground) was placed on the wrist of the left arm. The skin under the Ag/AgCl EMG-electrodes (Falk Minow Services) was cleaned with alcohol solution and scrubbed to reduce electrical impedance. An electrolytic gel was placed between the electrode surface and the skin to facilitate the conduction of the electrical signal. All electrodes were attached to the BrainAmp amplifier and signals were sampled by Brain Vision Recorder software with a sampling rate of 2,500 Hz/channel and a resolution of 0.1 μV (Brain Products GmbH, Munich, Germany).

#### Data analysis

EMG signals were processed by using MATLAB R2020b (Mathworks, Natick, USA) and EEGLAB v14.1.2 toolbox (Swartz Center for Computational Neuroscience, CA, USA, https://sccn.ucsd. edu/eeglab/index.php). At the beginning of the experiment, we recorded 3 maxima voluntary contractions (MVC) of the zygomatic muscle, in order to normalize the raw EMG signal and make the comparison of participants’ responses possible. Raw data were first normalized to MVC, band-pass filtered (20–250 Hz) with a 6th order Butterworth filter and rectified. Furthermore, we extracted EMG envelopes and divided the processed EMG signal in epochs of 8,000 ms, each corresponding to high, low and robotic emotional laughing stimuli (10 epochs for emotional laughing high, 10 epochs for emotional laughing low, and 10 epochs for emotional laughing control). A peak analysis was then performed. In particular, we calculated the zygomatic EMG activity around the peak of contraction (time window of ±1,200 ms around the peak), obtaining for each participant 3 curves of mean EMG peak activity (1 for emotional laughing high, 1 for emotional laughing low, and 1 for emotional laughing control) and then, considering all participants (group analysis), EMG curves corresponding to the same stimuli were aligned and averaged. Additionally, in order to highlight the effect of vitality forms during the emotional contagion phenomenon, the zygomatic activity related to high and low emotional laughing was normalized with that obtained during the control condition. In particular, the zygomatic activity high/low normalized (expressed in %) was calculated as zygomatic activity high/low ($\mu$V) *100/zygomatic activity control ($\mu$V). Finally, the EMG activity was correlated with the BOLD activity of subcortical areas showing a significant modulation during the processing of high and low emotional laughing (right amygdala, right thalamus, and periaqueductal gray, Fig. 4B).

## Results

### fMRI study

#### Brain activations during the observation of emotional laughing vs. control

In order to identify the neural substrates involved in the observation of vitality forms characterizing emotional laughing, we contrasted emotional laughing vs. emotional laughing control. This contrast showed bilateral cortical activations including part of the occipital lobe, especially the extrastriate body area (EBA) and the temporal pole. Additionally, activations were found in the mesial and dorsal premotor regions, especially on the right hemisphere (Fig. 2A1). At subcortical level, activations were found in the anterior part of the insula bilaterally, throughout almost the entire extent of the medial thalamus, mainly on the right side, in the anterior and ventral part of the putamen, in the posterior part of the caudate, in the amygdalae, especially in the right one, in the periaqueductal gray/raphe nucleus, and in the cerebellum (for coordinates and statistical values see Table 1). Notably, there was an activation of the left anterior insula involving the anterior and the middle short gyri (Fig. 2A2). In order to evaluate the BOLD activity in the left antero-ventral insula a region of interest (ROI) was defined centering a sphere (radium 3 mm) around the maxima (*x*-36 *y* 10 *z*-8) by using SPM MarsBaR Toolbox (release 0.42). Then, using the SPM Rex Toolbox, in this ROI the BOLD signal change was extracted. In this region, t test revealed a significant difference between the processing of high and low conditions (Fig. 2A2).

**Table 1:** Brain activations obtained during the direct contrasts emo laugh vs. control (A), non emo laugh vs. control (B), emo laugh vs. non emo laugh (C), non emo laugh vs. emo laugh (D). Local maxima, as shown in Figures 2 and 3 are given in MNI standard brain coordinates. Significant threshold was set at PFWE < 0.05 (cluster-level).

Contrast of interest	Left hemisphere					Right hemisphere			
	*x*	*y*	*z*	*Z*-score		*x*	*y*	*z*	*Z*-score
**(A) Emo Laugh vs. Control**									
Cerebellum	−12	−78	−20	6.49	Middle Temporal Gyrus	44	−66	2	Inf
Middle Occipital Gyrus	−44	−68	4	6.29	Lingual Gyrus	8	−76	−4	7.67
Middle Temporal Gyrus	−54	−56	8	4.56	Inferior Frontal Gyrus	38	28	−8	6.20
Insula	−36	10	−10	4.23	Premotor Cortex	42	12	38	6.10
					Posterior-Medial Frontal	4	24	54	5.60
					Cerebellar Vermis	0	−54	−42	5.59
					Thalamus	6	−10	8	5.54
					Superior Medial Gyrus	4	28	42	4.95
					Amygdala	26	−2	−12	4.70
					Periaqueductal Gray	6	−24	−8	4.54
					Cerebellum	8	−68	−36	4.05
					Angular Gyrus	34	−54	44	4.41
					IPS	42	−42	34	4.11
					Caudate	14	6	6	4.02
**(B) Non Emo Laugh vs. Control**									
Lingual Gyrus	−10	−78	−8	5.98	Lingual Gyrus	6	−74	0	7.72
Hippocampus	−24	−26	−10	5.68	Precentral Gyrus	44	6	48	5.79
Cerebellum	−10	−48	−48	4.49	Thalamus	8	−18	8	5.58
Inferior Frontal Gyrus	−42	12	26	3.96	Cerebellar Vermis	2	−56	−38	5.28
Insula	−36	10	−8	3.88	Fusiform Gyrus	42	−54	−22	5.15
					Middle Temporal Gyrus	56	−40	8	5.07
					Inferior Frontal Gyrus	38	16	28	4.73
					Cerebellum	44	−64	−24	4.60
					Insula	34	22	−4	3.83
**(C) Emo Laugh vs. Non Emo Laugh**									
Superior Temporal Gyrus	−42	−32	6	Inf	Superior Temporal Gyrus	52	−14	2	Inf
Cerebellum	−14	−64	−18	4.09	Heschis Gyrus	40	−24	8	7.17
					Middle Occipital Gyrus	40	−64	2	4.88
					Thalamus	2	−4	4	4.27
					Amygdala	24	4	−14	4.09
					Habenula	4	−26	10	4.00
					Temporal Pole	34	10	−26	3.89
					Periaqueductal Gray	0	−22	−12	3.39
**(D) Non Emo Laugh vs. Emo Laugh**									
Inferior Parietal Lobule	−44	−44	48	5.22					
Middle Temporal Gyrus	−54	−44	2	5.10					
Superior Frontal Gyrus	−22	−6	50	4.75					
Inferior Frontal Gyrus	−44	12	24	4.52					

Finally, the opposite contrast emotional laughing control vs. emotional laughing did not reveal any brain activation.

#### Brain activations during the observation of non-emotional laughing vs. control

The contrast non-emotional laughing vs. non-emotional laughing control revealed an activation pattern, at cortical and subcortical level, similar but weaker to that previously found in the contrast emotional laughing vs. emotional laughing control (Fig. 2B1). Notably, there was an activation of the left antero-ventral insula (Fig. 2B2). In order to evaluate the BOLD activity in this insular sector, a region of interest (ROI) was defined (*x*-36 *y*10 *z*-8) and the BOLD signal change was extracted. In this region, *t*-test analysis revealed a significant difference between the processing of high and low conditions (Fig. 2B3).

The opposite contrast non-emotional laughing control vs. non-emotional laughing did not reveal any brain activation.

#### Contrast between emotional laughing and non-emotional laughing

The contrast emotional laughing vs. non-emotional laughing showed activations of the superior temporal gyrus mainly in the right hemisphere (Fig. 3A). Most importantly, at subcortical level, activations were found in a series of centers known to be related to emotions: the right amygdala, the anterior thalamus/hypothalamus, the periaqueductal gray/raphe nucleus, the habenula/medial pulvinar, the right temporal pole (Fig. 3C). In order to evaluate the BOLD activity during the discrimination of high and low emotional laughing, a ROI analysis was carried out in all these regions (see also Supplementary Material). Specifically, 5 ROIs were defined: ROI1, right amygdala (*x* 24 *y* 4 *z*-14); ROI2, right thalamus (*x* 2 *y*-4 *z* 4); ROI3, periaqueductal gray (*x* 0 *y*-22 *z*-12); ROI4, right habenula (*x* 4 *y*-26 *z* 10); ROI5, right temporal pole (*x* 34 *y* 10 *z*-26). The analysis of the BOLD signal change extracted in all these ROIs revealed a significant difference between high- and low-vitality forms in ROI1, ROI2, and ROI3 (Fig. 4B).

In contrast, no significant difference was found in ROI4 and ROI5.

Finally, the opposite contrast non-emotional laughing vs. emotional laughing showed the activation of areas exclusively located in the left hemisphere (Fig. 3BD).

### EMG study

Results of the statistical analysis (paired *t*-test) revealed a significant difference of EMG signal relative to the discrimination of stimuli conveying high and low vitality forms (Fig. 4A2). In addition, we found a significant correlation between EMG and BOLD activities in the right amygdala, for both high (*R* = 0.61, *P* = 0.02) and low (*R* = 0.54, *P* = 0.04) emotional laughing (Fig. 4C).

## Discussion

### Observation of form and content of the emotional laughing

Rizzolatti and Sinigaglia (2016) have suggested that, besides cold actions, also those endowed with emotional content are expressed with different vitality forms. In this view, in the present study, we aimed to assess if the observation of vitality forms expressed in emotional behavior involved the same neural substrates associated to cold actions. For this purpose, an fMRI experiment was carried out. Specifically, participants were presented with emotional laughing and non-emotional laughing stimuli, each of them expressed with 2 different vitality forms (high and low). Both the observation of emotional and non-emotional laughing, with respect to their relative control conditions, produced the activation of the anterior-ventral insula. This insular sector corresponds to its anterior and middle short gyri, thus to a region adjacent but not overlapping with the dorso-central sector, known to be involved in the perception and execution of hand/arm vitality forms actions (Di Cesare et al. 2014, 2015, 2020, 2021). Our data showed that this sector is also activated during the execution of voluntary smile (see Supplementary Material) suggesting that it is endowed with mirror properties. Notably, this sector of the insula appears to correspond to that considered by Kurth and colleagues as involved in socio-emotional behaviors (Kurth et al. 2010). Moreover, our findings are in line with a previous study showing a similar insular activation during the observation of many types of emotional and neutral static faces (Wild et al. 2003). Interestingly, a ROI analysis carried out on brain areas involved in both emotional and non-emotional laughing observation showed that only in the left antero-ventral insula the BOLD activity associated to the high vitality form is significantly higher than that associated to the low one.

Besides the insula, the processing of emotional laughing produced the selective activation of areas known to be related to emotions: the right amygdala, the right temporal pole and, bilaterally, the anterior thalamus/hypothalamus, the habenula/medial pulvinar and the periaqueductal gray. A ROI analysis showed that in some of these structures (the right amygdala, right thalamus/hypothalamus, and periaqueductal gray), a significant modulation of the BOLD activity was present during the discrimination of high and low emotional laughing. These results indicate that, besides processing the emotional content, these subcortical sectors are also modulated by the form in which the emotion is expressed (high vs. low). It is important to note that, as already demonstrated by many neurological and functional studies regarding the emotional processing, the perception of emotional laughing mainly involved the right hemisphere (Gainotti 2021).

Considering the amygdala activity, it is well known its role in the perception of emotional facial expressions (Gothard 2014). A research based on 3 different approaches (behavioral, neuroimaging, and single-neuron recordings) showed that the human amygdala parametrically encodes the degree of emotion in facial expressions (Wang et al. 2017). These findings are completely in agreement with our results showing an increasing or decreasing of the BOLD activity in the right amygdala during the discrimination of high and low emotional laughing, respectively.

In conclusion, the here observed activation of the antero-ventral sector of the insula during of both the observation of emotional and non-emotional laughing, indicates that this insular sector is involved in the discrimination of an observed vitality forms regardless the presence or the absence of an emotional content.

### Emotional contagion

The observation of emotional laughing usually produces a spontaneous contagion, characterized not only by the activation of subcortical emotional centers, but in some subjects, also by specific behavioral responses, such as characteristic facial mimicry (De Gelder 2006). Although our fMRI study highlighted the neural substrates involved in the encoding of emotional laughing, it did not allow us to provide evidence of the motor activity related to an emotional contagion. To this aim, an EMG study was carried out on the same participants in a separate experimental session outside the scanner. Results showed that, during the discrimination of high and low emotional laughing, the modulation of the right amygdala activity found in the fMRI study corresponds to a modulation of the zygomatic muscles activity found in the EMG study. These results indicate that the discrimination of the different vitality forms is reflected by a correspondent muscles activity, i.e. the observation of high-emotional laughing produces a stronger zygomatic activity. Moreover, the amygdala activity is related not only to the discrimination of the emotional content and its form, but also to the motor response produced during the emotional contagion.

#### Circuits for processing emotional faces

The recognition of dynamic facial expression involves 2 main routes: a cortical route (dorsal stream for faces), consisting in the middle temporal area (MT) and its satellites selective for visual motion, among which the extrastriate body area (EBA), as well as the STS area(Furl et al. 2012; Bernstein and Yovel 2015), and a subcortical route, formed by the superior colliculus and the medial pulvinar (Gerbella et al. 2019). In our study, both these pathways were activated during the observation of emotional as well as non-emotional laughing, since in both conditions the observed faces were characterized by a clear dynamic component. Interestingly, both these pathways interact one to the other and have also a common target: the amygdala (Klingler and Gloor 1960; Amaral et al. 2003; Rafal et al. 2015), a structure that we described above to be selectively activated during the perception of emotional laughing.

It is noteworthy that in both conditions the classical parieto-premotor territories of the mirror neuron network are activated, especially in the right hemisphere in the emotional condition and in the left hemisphere in the non-emotional one. The stronger right hemisphere activation is in line with the aforementioned evidence that this hemisphere is crucial to process emotions (Gainotti 2021). In contrast, the stronger activation observed in the left hemisphere during the non-emotional condition seems to be related to the presence of verbal material. Finally, in both conditions but especially in the emotional one, a strong activation was found in the preSMA. Since this cortical area plays a crucial role in voluntarily modulating/inhibiting various typology of movements, among which laughter (Gerbella et al. 2021), its activity seems to reflect the instruction of not to laugh given to participants. A relatively surprising negative result is the lack of activation of the pregenual anterior cingulate cortex (pACC), a cortical territory activated during the production of laughter and during its perception (Caruana et al. 2015, 2018). A possible explanation of this result could be the role of this cortical region in the production of laughter during the emotional contagion rather than during its passive observation. This idea is in line with previous intracranial recording results on epileptic patients, according to which only in some subjects, likely only those in whom the emotional contagion evokes overt laughter, the observation of laughter in others produces an activation of this sector of the cingulate (Caruana et al. 2018). One could speculate that our subjects, that are instructed of not to laugh, as well as the unresponsive patients of Caruana et al. (2018) have a top-down inhibition of facial expressions. A recent study in which close areas of the medial prefrontal cortex, among which pre-SMA, have been found linked to reduced smile mimicry in a concurrent fMRI and facial EMG study supports this hypothesis (Korb et al. 2019). Summarizing, the instruction of not to laugh given to participants should explain not only the activation of the pre-SMA but also, throughout a top-down inhibition, the lack of the pACC activity.

## Conclusions and limitations

In conclusion, the present study provides evidence that both emotional and non-emotional laughing, conveying different vitality forms, activate the insula, suggesting its role in the processing of vitality forms information regardless the presence or the absence of an emotional content. Additionally, we showed that the observation of emotional laughing produces the activation of a series of brain regions, known to be related to emotions. Among these areas, the right amygdala is the only one in which, during the observation of emotional laughing, its BOLD activity significantly correlates with the zygomatic muscles activity recorded during the EMG study.

Some potential limitations of the current study should be considered. First, the sample size was relatively small decreasing its representation of the entire population. Second, since the emotional stimuli were characterized by both visual and acoustical information, the activation of the amygdala during the observation of these stimuli remains unspecified and cannot be ascribed to visual, acoustical, or both information. Further experiments will address this issue in the future. Third, due to problems of signal noise during the EMG recordings inside the scanner, the EMG experiment was carried out separately from the fMRI study.

## Authors’ contributions

G.D.C., M.G., G.L., A.S., and G.R. contributed to the study design. G.D.C. and G.L. conducted the experiments. G.D.C., G.L., and M.M. performed data analysis. G.D.C, G.L, M.G., A.S., and G.R. edited the manuscript and approved the final version of the manuscript for submission.

## Supplementary Material

Supplementary_final_bhac334
