# Supplementary material for: Investigating form and content of emotional and non-emotional laughing
Source: Cereb Cortex. 2022 Sep 9;33(7):4164–72. doi: 10.1093/cercor/bhac334 (PMC10068279; doi:10.1093/cercor/bhac334)
Supplement: Supplementary_final_bhac334 [file supplementary_final_bhac334.docx]

**Supplementary Material**

*Preliminary behavioral study*

A preliminary behavioral study, conducted before the fMRI experiment, was carried out in order to ascertain that the high and low intensities of both emotional and non-emotional stimuli were really perceived as different. Thirty healthy right-handed participants (16 females and 14 males, mean age=25.3 years, SD=2.87 years) took part in this behavioral study. Participants were presented with video-clips showing an actor and an actress laughing (*emotional laughing*) or requiring to laugh (*non-emotional laughing*), with two different intensities (high and low), or a robot performing the same laugh (*emotional laughing control*) or request (*non-emotional laughing control* in a robotic way. In total 18 stimuli were presented: 8 stimuli for the *emotional laughing* condition, 8 for the *non-emotional laughing* condition, 1 for the *emotional laughing control* condition and 1 for the *non-emotional laughing control* condition. During the experiment, participants were required to perceive the stimuli and then evaluate, in terms of sound and facial expression, their intensity (corresponding to vitality forms) by using a five points likert scale: very low (0-20%), low (20%-40%), neutral (40%-60%), high (60%-80%), very high (80%-100%). Participant’s responses were analyzed by using two GLM models, the first for *emotional laughing* condition and the second for *non-emotional laughing* condition. Results of the first GLM (Figure S1A) showed a significant difference among high, low and robotic stimuli (p<0.001). Post hoc analysis (Bonferroni correction) revealed that high and low vitality forms were perceived as significantly different (p<0.001). Moreover, the perception of the robotic stimuli significantly differs from that of high and low vitality forms (p<0.001). Results of the second GLM (Figure S1B) showed a significant difference among high, low and robotic stimuli (p<0.001). Post hoc analysis (Bonferroni correction) revealed that high and low vitality forms were perceived as significantly different (p<0.001). Moreover, the perception of the robotic stimuli significantly differs from that of high and low vitality forms (p<0.001). On the basis of these results, the same stimuli were used in the following fMRI experiment.

**
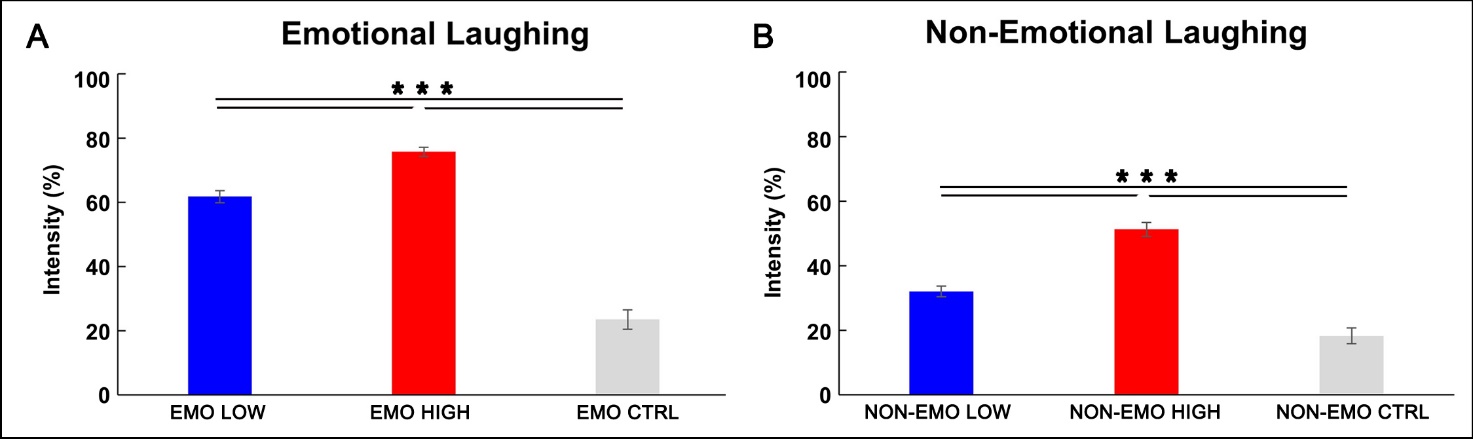
Figure S1: Results of the first GLM, showing a significant difference (***p<0.001, Bonferroni correction) among high, low and robotic *emotional laughing* stimuli (A). Results of the second GLM, showing a significant difference (***p<0.001, Bonferroni correction) among high, low and robotic *non-emotional laughing* stimuli (B). Labels on the y-coordinate refer to the five points of the likert scale and correspond to percentage values of intensity: very low 0%-20%; low 20%-40%; neutral 40%-60%; high 60%-80%; very high 80%-100%.**

*fMRI data acquisition and pre-processing*

Anatomical T1-weighted and functional T2*-weighted MR images were acquired with a 3 Tesla General Electrics scanner equipped with an 8-channel receiver head-coil. Functional images were acquired using a T2*-weighted gradient-echo, echo-planar (EPI) pulse sequence acceleration factor asset 2, 40 sequential transverse slices (slice thickness = 3 plus inter-slice gap = 0.5 mm) covering the whole brain, with a TR time of 3000ms (TE = 30ms, flip-angle = 90 degrees, FOV = 205 x 205 mm^2^, in-plane resolution 2.5 x 2.5 mm^2^). The scanning sequence comprised 280 sequential volumes. Additionally, a T1 weighted structural image was acquired for each participant (acceleration factor arc 2, 156 sagittal slices, matrix 256x256, isotropic resolution 1x1x1 mm^3^, TI=450ms, TR =8100ms, TE = 3.2ms, flip angle 12°). Data analysis was performed with SPM12 (Wellcome Trust Center for Neuroimaging, London, UK) running on MATLAB R2020b (The Mathworks, Inc.). For each subject, all volumes were slice timing corrected, spatially realigned to the first volume of the first functional run and unwarped to correct for between-scan motion. For all subjects, head motion was carefully checked along x (pitch movement), y (yaw movement) and z (roll movement) directions and no participant has met the exclusion criteria of 3 mm mean displacement (translation > 3mm or rotation > 3°). T1-weighted image was segmented into gray, white and cerebrospinal fluid and spatially normalized to the Montreal Neurological Institute (MNI) space. Spatial transformation derived from this segmentation was then applied to the realigned EPIs for normalization and re-sampled in 2×2×2 mm^3^ voxels using trilinear interpolation in space. All functional volumes were then spatially smoothed with a 6-mm full-width half maximum isotropic Gaussian kernel. The experiment consisted in 3 functional runs with a total of 6 blocks for each condition. Each functional run lasted about 10min. Finally, in order to identify the sector of the insula involved in execution of a smile, we carried out also a brief localizer run. Specifically, participants were required to perform a voluntary smile without moving the head. In total each participant performed 24 smiles.

*Brain activations during the perception of Emotional Laughin*g *vs. Baseline*

During the *emotional laughing* condition, participants observed an actor or an actress who spontaneously laughed and listened to the respective laughing sound. Notably, the actors laughing was produced with two different intensities, high and low, corresponding to two different vitality forms. During this task, participants were required to perceive the audio-visual stimuli without laughing. The reason for this instruction was to avoid an overt motor component. The contrast *emotional laughing vs. baseline* showed bilateral activations extending from the occipital lobe, involving particularly the extrastriate body area (EBA), to the posterior part of the temporal gyrus. Additionally, activations were also found in the mesial and dorsal premotor regions, especially on the right hemisphere (Figure S2A). At subcortical level, activations were found in the anterior part of the insula bilaterally, throughout almost the entire extent of the medial thalamus, mainly on the right, in the anterior and ventral part of the putamen, in the posterior part of the caudate, in the amygdalae, especially in the right one, in the colliculi, mainly in the right one, in the periaqueductal grey/raphe nucleus, and in the cerebellum. During the *emotional laughing control* condition, participants observed a humanoid robot (iCub) who laughed in a “robotic” neutral way (i.e. without any vitality form). The contrast *emotional laughing* *control vs.* *baseline* showed activations involving occipital visual areas and the superior temporal gyrus, thus involving purely visual and acoustic areas. (Figure S2B).

*Brain activations during the perception of Non-Emotional Laughing vs. Baseline*

During the *non-emotional laughing* condition, participants observed the same actors performing a smile and simultaneously pronouncing the Italian verb “ridi” (English version: “laugh”), with two different intensities (high and low) corresponding to two different vitality forms). During this task, participants were required to perceive the audio-visual stimuli but not to execute the requested action (laughing). The reason for this instruction was to avoid an overt motor component. The contrast *non-emotional laughing* *vs*. *baseline* enhanced the activation of the occipital lobe, including the EBA area, the entire superior temporal gyrus and the mesial and dorsal premotor cortex bilaterally. In addition, in the left hemisphere, there was an extensive activation of the inferior frontal gyrus and an activation of the inferior parietal lobe (Figure S2C). At subcortical level, activations were found in the anterior insula bilaterally, throughout medial thalamus, mainly on the right, in the anterior and ventral part of the putamen, in the posterior part of the caudate, in the colliculi, in the periaqueductal grey, and in the cerebellum. In the control condition (*non-emotional laughing control*), participants observed the humanoid robot performing a smile and simultaneously pronouncing the same verb (“ridi”) in a “robotic” neutral way (i.e. without any vitality form). The contrast *non-emotional laughing control* *vs.* *baseline* revealed the bilateral activation of the occipital cortex and the superior temporal gyrus, the inferior frontal gyrus and the inferior parietal lobe in the left hemisphere (Figure S2D).


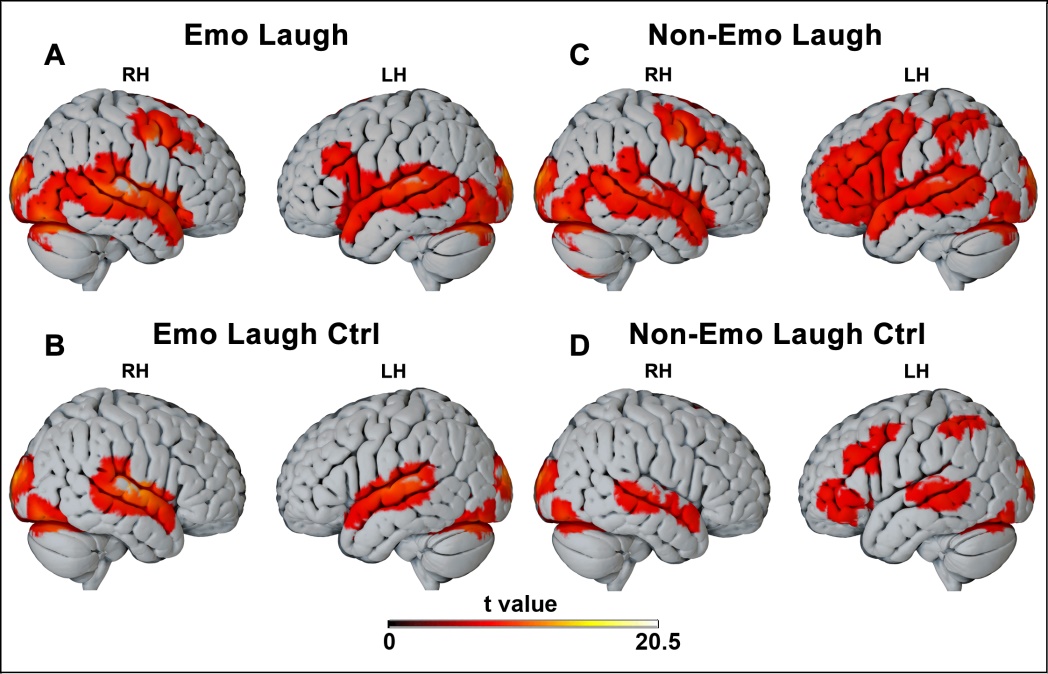
**Figure S2. Brain activations resulting from the *emotional laughing vs. baseline* contrast (A), the *non-emotional laughing vs. baseline* contrast (B), the *emotional laughing control vs. baseline* contrast (C), the *non-emotional laughing control vs. baseline* contrast (D). These activations are rendered using a standard Montreal Neurological Institute Brain Template (P_FWE_<0.05 at cluster level).**

*Conjunction and ROIs Analyses*

In order to highlight brain regions involved in both emotional and non-emotional laughing perception versus their relative controls, a conjunction analysis was performed. Results of this analysis revealed the activation of PMv and preSMA areas in the right hemisphere, pSTS and insula bilaterally. Furthermore, in all these regions, a ROI analysis was carried out to assess the BOLD activity relative to the processing of high and low vitality forms. In particular, six ROIs were defined centering a sphere (radium 3mm) around the maxima by using SPM MarsBaR Toolbox (release 0.42) (ROI 1: right PMv, x 42 y 12 z 32 ; ROI 2: right pSTS, x 52 y -54 z 14; ROI 3: right insula, x 40 y 18 z -10; ROI 4: right preSMA, x 6 y 8 z 56; ROI 5: left insula, x -36 y 10 z -8; ROI 6: left pSTS x -50 y -54 z 8). Results revealed a significant difference between high and low vitality

forms, for both emotional and non-emotional laughing perception, only in the left insula (Figure S3B, panel 5).

*
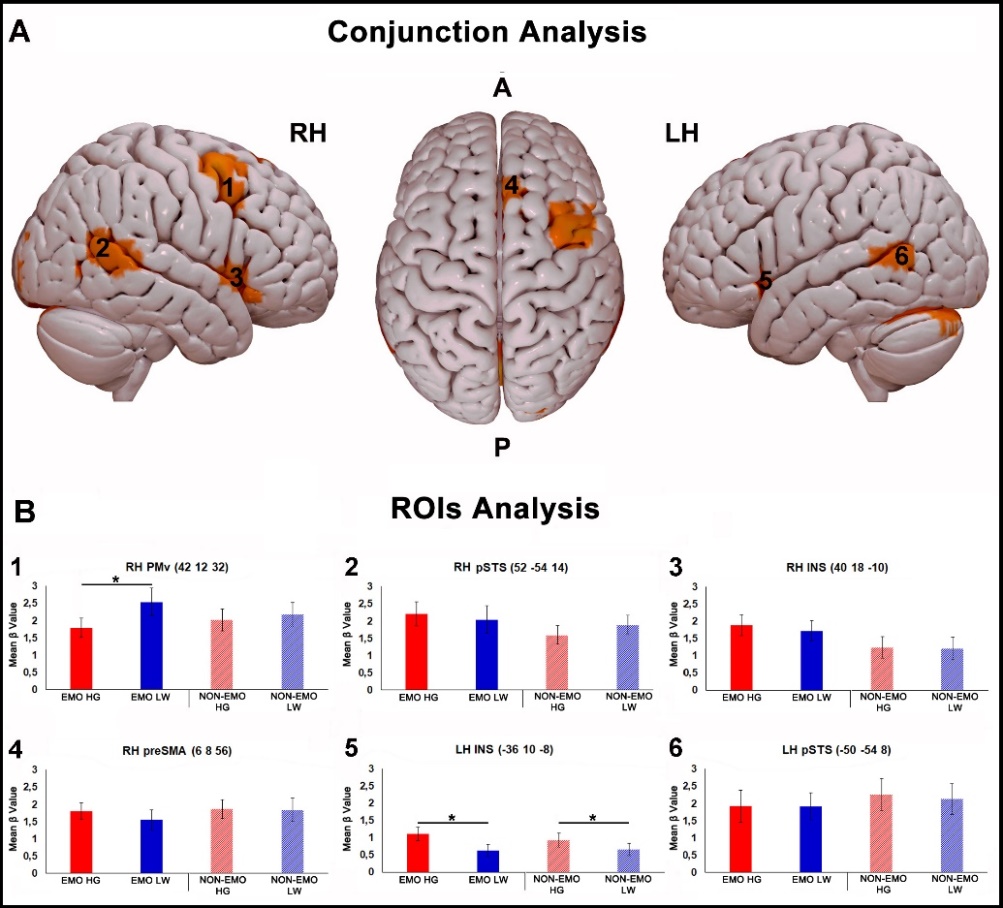
***Figure S3: Brain activations resulting from the conjunction analysis *emotional laughing* *vs*. *emotional laughing control* & *non-emotional laughing vs. non-emotional laughing control* (A). Signal changes in ROIs created on the right PMv (RO1), right pSTS (ROI2), right insula (ROI3), right preSMA (RO4), left insula (ROI5) and left pSTS (ROI6). The horizontal lines above the columns indicate the comparisons between high and low vitality forms. These activations are rendered using a standard Montreal Neurological Institute Brain Template (P_FWE_<0.05 at cluster level). *Significant differences (p ≤ 0.05).**

*Brain activations during the execution of Non-Emotional Laughin*g *vs. Baseline*

During the execution of *Non-Emotional Laughin*g (localizer run), participants were required to perform a voluntary smile. The contrast *Non-emotional laughing* *execution* *vs*. *baseline* enhanced the activation of the inferior parietal lobe, the ventral part of the motor area and the ventral part of the premotor cortex extending to the inferior frontal gyrus and the insula. Notably, all these activations were bilaterally (Figure S4).


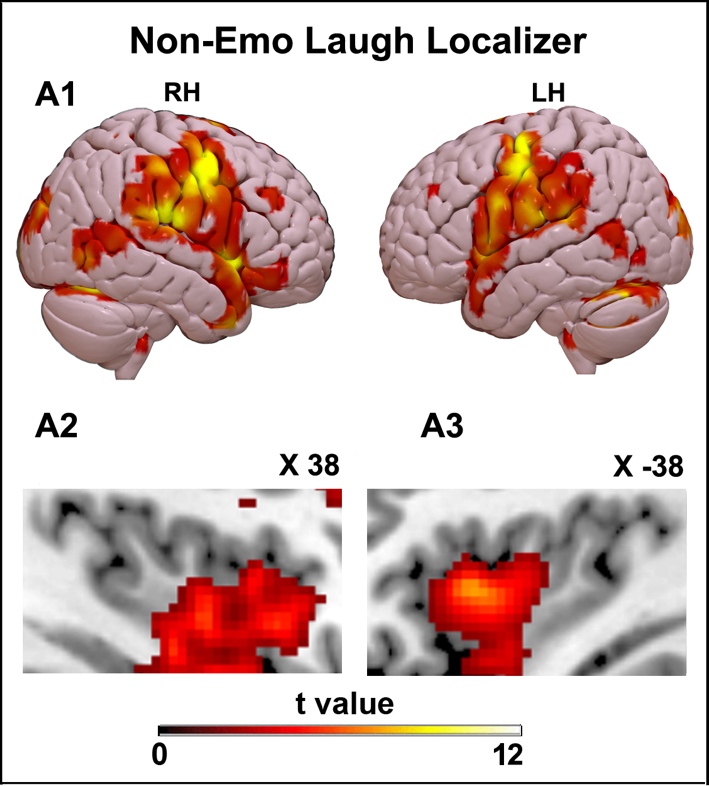


**Figure S4: Brain activations resulting from the localizer run (contrast: *non-emotional laughing execution* *vs*. *baseline)* (A1). Activations of the left and right insula obtained in this contrast are shown in panels A2 and A3 respectively. All These activations are rendered using a standard Montreal Neurological Institute Brain Template (P_FWE_<0.05 at cluster level). *Significant differences (p ≤ 0.05).**
